# Supplementary material for: Asymmetric Synthesis of Quaternary Hydantoins via a Palladium-Catalyzed Aza-Heck Cyclization
Source: J Am Chem Soc. 2025 Nov 14;147(49):44692–8. doi: 10.1021/jacs.5c16022 (PMC12703750; doi:10.1021/jacs.5c16022)
Supplement: Supplementary file 2 [file ja5c16022_si_002.zip › All NMR FID Files/27/27_AllNMR/1/pdata/1/email_EM05-028-1_1_1.pdf]

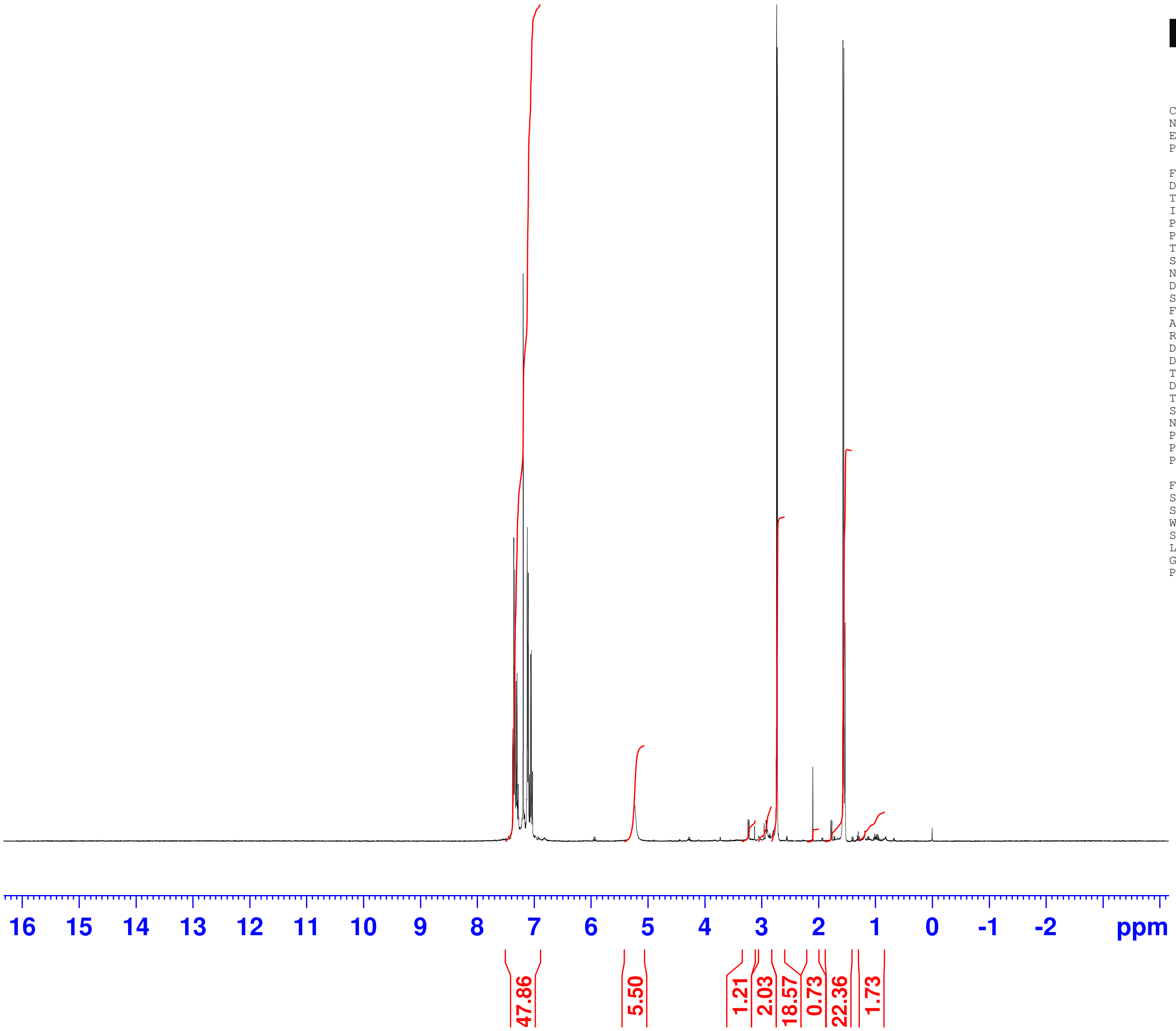

BRUKER

```
Current Data Parameters
NAME           EM05-028-1
EXPNO                   1
PROCNO                  1

F2 - Acquisition Parameters
Date_            20231129
Time                11.30 h
INSTRUM   Avance Neo 400 U of DE Brown Lab
PROBHD    Z104450_0412 (
PULPROG              zg30
TD                  65536
SOLVENT             CDCl3
NS                     16
DS                      2
SWH              8196.721 Hz
FIDRES           0.250144 Hz
AQ              3.9976959 sec
RG                    101
DW                 61.000 usec
DE                  12.69 usec
TE                  298.0 K
D1             1.00000000 sec
TD0                     1
SFO1          400.1324708 MHz
NUC1                   1H
P0                   5.00 usec
P1                  15.00 usec
PLW1           8.31639957 W

F2 - Processing parameters
SI                  65536
SF            400.1300366 MHz
WDW                    EM
SSB                     0
LB                   0.30 Hz
GB                      0
PC                   1.00
```
